# ET-1, MMPs, ZAG, and APN Link Reduced Ocular Perfusion to Glaucoma

**DOI:** 10.3390/biom15101364

**Published:** 2025-09-25

**Authors:** Maren Kasper, Kai Rothaus, Lasse Schopmeyer, Dirk Bauer, Swaantje Grisanti, Carsten Heinz, Karin Loser, Claudia Lommatzsch

**Affiliations:** 1 Department of Ophthalmology and Ophtha-Lab at St. Franziskus-Hospital, 48145 Muenster, Germany; 2 Department of Anaesthesiology, Erasmus Medical Center (EMC), 3015 GD Rotterdam, The Netherlands; 3 Department of Ophthalmology, University of Luebeck, 23562 Luebeck, Germany; 4 Department of Ophthalmology, University of Essen, 45147 Essen, Germany; 5 Department of Human Medicine, University of Oldenburg, 26129 Oldenburg, Germany

**Keywords:** OCT angiography, glaucoma, endothelin, cytokines, ocular perfusion, vessel density, POAG, XFG, biomarker

## Abstract

**Purpose**: This study sets out to analyze the correlation of ET-1, a vasoactive peptide, along with various cytokines and vascular factors, with clinical parameters and OCT/OCT-A measurements in glaucoma participants. **Methods:** Eyes of participants with cataract (*n* = 30) or glaucoma (*n* = 87) were examined with optical coherence tomography (OCT) and OCT angiography (OCT-A). Aqueous humor (AqH) from the examined eye and plasma were sampled during cataract or glaucoma surgery and analyzed by means of ELISA and Luminex assay to determine their levels of ET-1 and 35 proteins deemed relevant for regulation of the AqH outflow pathway, ocular perfusion (OP), and glucose metabolism. **Results:** Glaucomatous eyes are characterized by reductions in RNFL thickness and OP, reflected by reduced vessel density. Furthermore, significantly elevated peripheral ET-1 levels were detected in participants with glaucoma. In addition, significantly elevated AqH levels of MMP-2, MMP-3, ET-1, sEMMPRIN, ZAG, sLOX-1, follistatin, cortisol, endostatin, sTIE-2, and PDGF-BB were detected in the glaucomatous eyes, with correlation to reduced VD for APN, C3a, MMP-3, resistin, sTIE-2, and ZAG. Multivariable analysis showed a correlation of AqH APN levels with the reduced VD in glaucomatous eyes. **Conclusions:** The peripheral ET-1 level and the intraocular levels of APN, C3a, MMP-3, resistin, sTIE-2, and ZAG are associated with impaired OP in glaucoma. Furthermore, elevated intraocular levels of MMP-3, ZAG, and APN were identified as biomarkers for impaired perfusion in glaucoma.

## 1. Introduction

Glaucoma, a progressive optic neuropathy [1], is characterized by the degeneration of retinal ganglion cells (RGCs). This degeneration leads to irreversible visual field loss and can culminate in blindness [2].

The primary risk factor for the development of glaucoma is elevation of intraocular pressure (IOP), which is determined by the balance between the production of aqueous humor (AqH) by the ciliary body epithelium and its outflow through the trabecular meshwork, Schlemm’s canal, or the uveoscleral tract. In glaucomatous eyes, AqH drainage is often compromised, resulting in heightened IOP and subsequent damage to the optic nerve [3]. Traditional perimetry has substantial limitations in early disease detection, as functional deficits on visual field testing typically become apparent only after approximately 40% of RGCs have been lost [4]. Studies have demonstrated that perimetric changes lag behind structural damage, with perimetry detecting changes approximately four years after retinal nerve fiber layer (RNFL) thinning becomes apparent on imaging, and potentially eight years after the onset of early RGC dysfunction as detected by electrophysiology [5,6]. Additionally, perimetry results are limited by test variability [7], making it difficult to assess progression, particularly in early disease stages. Non-invasive techniques such as optical coherence tomography (OCT) have emerged as useful tools for identifying RGC loss [8] and monitoring the RNFL. However, even OCT is not without limitations; the so-called floor effect can hinder accurate assessment of advanced stages of glaucoma [9]. Hence, additional parameters and biomarkers are sought. Studies have explored the role of ocular perfusion (OP), including endothelial dysfunction and impaired perfusion, together with systemic factors, in glaucoma progression [10,11]. OCT-angiography (OCT-A) can be used to monitor glaucoma-related reduction in OP [12]. In this context, impaired perfusion in glaucoma is reflected by reduced vessel density (VD), as defined by the device manufacturer to describe perfusion parameters, in the papillary and macula area [13,14,15,16]. Several studies have suggested a relationship between elevated plasma levels of endothelin-1 (ET-1) and glaucoma progression [17,18,19,20,21]. ET-1, a potent vasoconstrictor, has been shown to be associated with compromised OP, which underscores its potential as a biomarker in glaucoma [13]. Further studies have investigated the cytokine profile in the AqH of glaucomatous eyes, revealing their possible role in the disease’s pathogenesis [22,23,24]. However, no studies have investigated a possible correlation among ET-1, the altered cytokine profiles in glaucomatous eyes, and the altered OP [25,26,27,28]. This study addresses the correlation between ET-1, the altered cytokine pattern, and OP in glaucomatous eyes with the aim of elucidating the pathophysiological mechanisms of glaucoma and identifying critical biomarkers and therapeutic targets to advance its management.

## 2. Materials and Methods

### 2.1. Study Design

This prospective, single-center study was conducted at the Department of Ophthalmology and Ophtha-Lab of St. Franziskus-Hospital Muenster (Germany). The study was approved by the ethics committee of the Medical Association of Westfalen-Lippe, Germany (reference number 2021-670-f-S; approval date 10 November 2021) and adhered to the tenets of the Declaration of Helsinki. Prior to the study entry, all patients provided written informed consent. The patient recruitment took place between 2018 and 2020.

### 2.2. Subjects

The people included in the study were participants (age ≥ 18 years) either with glaucoma or without glaucoma (control group) with a medical indication for surgery (control group: cataract surgery; glaucoma group: cataract or glaucoma surgery). Participants were required to be ≥18 years of age, as OCT and OCT-A manufacturers do not provide pediatric normative databases, and RNFL thickness and vascular perfusion parameters in children differ significantly from adult values, precluding valid comparisons with our adult control group.

### 2.3. Examinations

Participants underwent a comprehensive ophthalmic examination (without pupil dilation) to assess their best-corrected visual acuity in LogMAR, slit-lamp biomicroscopy with indirect ophthalmoscopy, and measurement of mean arterial pressure (MAP).

Cataract assessment was performed using slit-lamp biomicroscopy. Lens opacities were clinically graded based on the Lens Opacities Classification System III (LOCS III) criteria [29]. OCT imaging was performed using SD-OCT (RTVue-XR; Optovue, Inc., Fremont, CA, USA—software version 2016.2.035). The following parameters were measured: RNFL thickness, ganglion cell complex (GCC) thickness, focal and global loss volume, CDR, and rim volume. Finally, IOP was measured by Goldmann applanation tonometry.

The diagnosis of glaucoma was based on the results of funduscopy and OCT. **Inclusion criteria** for the glaucoma group were as follows (at least two criteria had to be fulfilled): increased vertical cup-to-disc ratio (VCDR; ≥0.5); VCDR asymmetry > 0.2; and/or glaucomatous reduction in peripapillary RNFL and/or GCC thickness at the posterior pole.

The control group comprised participants meeting the following inclusion criteria: IOP ≤ 21 mmHg, no history of elevated IOP, and no positive family history of glaucoma. Clinically, the optic disc had to appear normal, characterized by an intact neuroretinal rim, unremarkable RNFL and GCC analysis, and VCDR asymmetry of <0.1.

The **exclusion criteria** for both groups were as follows: any ocular disease apart from mild to moderate cataract in the glaucoma group, or refractive error > ±6 diopters (D) sphere and ± 2D cylinder. Image/quality was deemed to be inadequate with an OCT-A signal strength index of <45 and/or scan quality < 6. Furthermore, patients with previous surgical treatment apart from selective laser trabeculoplasty/iridotomy (<6 months before inclusion) were excluded. Additionally, patients with a history of systemic vascular disease (diabetes mellitus, arterial hypertension, status post apoplexy/myocardial infarction/thrombosis, etc.) or a history of systemic vasoactive medication were also excluded.

OCT-A imaging was performed using AngioVue™—RTVue-XR (Optovue, Fremont, CA, USA, software version 2016.2.035). All scans were taken centered on the fovea (6 × 6 mm) and on the optic disc (4.5 × 4.5 mm). To ensure accurate centration and segmentation, all OCT-A images were reviewed by the same person (CL). Images of insufficient quality (e.g., motion artifacts despite the use of 3D projection artifacts removal, blurring, signal strength index < 45, or scan quality < 6) were excluded.

The optic disc scan was conducted in the segmentation area of the superficial nerve fiber layer, as designated by the manufacturer as the “radial peripapillary capillaries” (RPC). This area extends from the internal limiting membrane (ILM) to the posterior border of the RNFL. The VD was calculated automatically by the software. The VD (in %) was evaluated in three ways: as a total value of the peripapillary zone (ONH Whole), as an average peripapillary VD value (PeriONH Average), and by considering seven sectors (PeriONH NasSup, NasInf, InfNas, InfTemp, TempInf, TempSup, SupTemp, and SupNas).

In the macular region, segmentation was performed into the superficial vascular plexus (SVP) and the deep vascular plexus (DVP), as defined by the manufacturer: The SVP was defined as the interval between the inner limiting membrane (ILM) and the inner plexiform layer (IPL), with a depth of −10 µm. The DVP was defined as the interval between the IPL and the outer plexiform layer (OPL), with a depth of +10 µm.

For both segmentations, the VD values (in %) were included in the evaluation, in addition to an overall value (Macula Whole) for the following regions: foveal area (Fovea, diameter of 1 mm), parafoveal, (ParaFovea Average, diameter of 3 mm), and perifoveal (PeriFovea Average, with a diameter of 6 mm), with each region divided into four quadrants (Temp, Sup, Nas, and Inf).

Furthermore, the foveal avascular zone (FAZ; in mm^2^) was calculated as a combination of the two plexuses and was referred to by the manufacturer as the “retinal” segmentation layer (ILM to OPL +10 µm) with additional consideration of the acircularity index (AI).

In accordance with the IntRIS consensus recommendations (Munk et al., 2025) [29], we use the term ‘perfusion’ rather than ‘blood flow’ when describing OCT-A findings, as OCT-A detects motion contrast from moving red blood cells rather than directly measuring flow velocity. Vessel density measurements serve as validated surrogate markers for perfusion status [30]. The device manufacturer (Optovue) uses the term ‘vessel density (VD)’ to quantify the percentage of area occupied by perfused vessels in their software output. We have retained this manufacturer-specific terminology when reporting measured values to ensure consistency and reproducibility, while using ‘ocular perfusion (OP)’ in the general text to accurately reflect the physiological parameter being assessed.

### 2.4. Glaucoma Staging

Glaucoma severity was classified using the OCT Glaucoma Staging System (OCT GSS), which plots two OCT parameters on an x–y diagram to simultaneously provide a standardized classification of RNFL defect severity (healthy, border, stages 1 to 5) and the defect localization [31].

### 2.5. Peripheral Blood and Intraocular Samples

AqH (100 to 150 µL) and venous blood (heparin blood; 7 mL) were collected during the anesthetic period of planned glaucoma or cataract surgery. The blood was centrifuged at 2000× *g* for 10 min at 4 °C. AqH and plasma were transferred to 1.5 mL Eppendorf tubes and stored for up to 2 years at −80 °C until further analysis.

### 2.6. Protein Quantification

Protein levels in AqH and plasma samples were analyzed using a commercially available ET-1 Quantikine ELISA Kit (RnD Systems, Minneapolis, MN, USA). Furthermore, the cytokines adiponectin (APN), angiogenin, angiopoietin-1, angiostatin, arginase-1, bone morphogenetic protein (BMP)-2, BMP-9, BigET-1, C3a, creatine kinase myocardial band (CKMB), ciliary neurotrophic factor (CNTF), cortisol, soluble extracellular matrix metalloproteinase inducer (sEMMPRIN), endoglin, endostatin, soluble E-selectin (sE-selectin/CD62E), follistatin, interleukin(IL)-6, IL-8 (CXCL8), soluble lectin-like oxidized low-density lipoprotein (LDL) receptor-1 (sLOX-1), monocyte chemotactic protein 1 (MCP-1/CCL2), MHC class I polypeptide-related sequence B (MICB), MMP-2, MMP-3, MMP-9, N-terminal pro b-type natriuretic peptide (NTproBNP), platelet-derived growth factor beta polypeptide B (PDGF-BB), placental growth factor-1 (PlGF-1), resistin (ADSF), serum amyloid A (SAA), survivin (BIRC5), sTIE-2, tumor necrosis factor-α (TNF-α), vascular endothelial growth factor A (VEGF-A), and zinc alpha 2-glycoprotein (ZAG) were quantified using a customized Luminex kit (35plex, Life Technologies GmbH, Darmstadt, Germany). The Luminex assay was measured in a Bio-Plex MAGPIX Multiplex Reader (BioRad, Hercules, CA, USA). The cortisol assay was analyzed via Bioplex Manager 6.1 software (Biorad); other target proteins were quantified using Procartaplex Analyst V1.0 software. Standards and samples were measured in duplicates according to the manufacturer’s instructions. To visualize the significant differences between the glaucoma and cataract cohorts volcano plots were generated with VolcaNoseR [32].

### 2.7. Statistical Analysis

All statistical analyses were conducted using R Version 4.3.2 (31 October 2023; Dormagen, Germany). Data were tested for normal distribution by means of the Shapiro test. For samples with more than 30 observations or for normally distributed observations (parametric), the mean and standard deviation were used to express the data. In the absence of a normal distribution, the median and the interquartile range (25% and 75%) were reported (non-parametric). The significance level was set at α = 5%. A two-sample *t*-test was used to compare the means of two groups, provided that the requisite assumptions were met. As a non-parametric alternative, a Wilcoxon rank-sum test (Mann–Whitney U test) was used. To compare nominally scaled data, the chi-square test was applied on a 2 × 2 contingency table with each cell containing at least five entities; otherwise, Fisher’s exact test was used.

Exclusion and recoding of cytokine variables: Cytokines with concentrations above detectability level in fewer than five samples were excluded from the statistical analysis. For cytokines where all values above 0 were identified as outliers (following the 1.5∙IQR outlier rule), an additional dichotomous variable was introduced and recoded as “cytokine level > 0”.

Descriptive correlation analysis: To enable the use of generalized correlation analysis, generalized linear models were applied. If the second variable was at least interval-scaled, a common linear model was applied. Logistic regression (quasibinomial family) was used for dichotomous variables and multinomial regression for nominal variables with more than two attributes. To reduce the influence of outliers, weights were introduced for regression models using the 10% ($p_{10}$) and 90% percentiles ($p_{90}$)

$$w\left(x\right)=\exp-0.5\cdot {\left(\frac{\max\left\{0,\;t_{low}-x,\;x-t_{high}\right\}}{0.5\cdot \left(t_{high}-t_{low}\right)}\right)}^{2},where\;$$

$$t_{low}=p_{10}-1.5\cdot \left(p_{90}-p_{10}\right)\;and\;t_{high}=p_{90}+1.5\cdot \left(p_{90}-p_{10}\right)$$

To measure the acuity of the model, the McFadden pseudo-R^2^ measure was calculated [33], and a correlation index was derived by $\rho =sgn\left(\beta \right)\sqrt{R^{2}}$, where β is the model estimate.

Relationships with a rho (ρ) of 0.10 or less were considered irrelevant. In this research, rho and *p*-value were not combined, and it is advisable to avoid using the term “significance” in this context. A rho-value of 0.30 corresponds to an average effect, while a rho-value of 0.50 corresponds to a strong effect.

After outlier removal by the 1.5∙IQRmethod, multivariate analysis was applied in the following steps:

(1) Selection of variables: Constant variables, variables with less than 66% data, and attributed variables with less than seven samples per category were discarded.
(2) Data imputation: For model selection the data were imputed by means of the MICE algorithm [34]. After computing the pseudo-R^2^ matrix of all independent variables, we applied hierarchical clustering to separate these variables to k = max (3, (*n* − 50)/8) clusters. To avoid overfitting, only one variable per cluster was used to predict missing values for each independent variable.
(3) Selection of covariates: We used 100 times of fivefold cross-validation of Lasso variable selection and discarded the variables which were selected in fewer than 5% of runs. Finally, using the median penalty weight factor lambda, the variables were selected by a concluding Lasso process using glmnet [35,36] from the complete data. For the Lasso approach, we allowed a slightly overfitted model (*n* ≥ 35 + 6.5∙m, with sample size m and model size m) to reduce the risk of missing important variables.
(4) Model reduction: Since the Lasso approach does not account for *p*-values but selects variables to maximize a weighted sum of log-likelihood and model penalty, we performed iterative backward selection to discard the most insignificant variable of the multivariable model, and thus fulfill the predefined model size condition (*n* ≥ 50 + 8∙m) [37]. Model size limitation is important to avoid overfitting of models, which reduces the statistical power and increases risk of finding random correlations.
(5) Application of the model to original data: Finally, the model resulting from step 4 was applied on the original, non-imputed data. The most insignificant variable was iteratively dropped until the model size condition was fulfilled on these data.

All *p*-values were adjusted by the Benjamini–Hochberg method [38] to counteract the multiple testing problem.

## 3. Results

### 3.1. Participants

The subjects included in the current study were previously characterized in depth with regard to glaucomatous defects by means of the OCT staging tool, OCT-A analysis, and ET-1 quantifications in samples of AqH and plasma [13]. In addition to the patient cohort analyzed in the previous study, a further 17 glaucomatous eyes were evaluated. A total of 117 eyes from 107 participants were therefore included in the present study (Table 1). 87 of these were glaucomatous eyes, subdivided into POAG (*n* = 63), pseudoexfoliation syndrome (XFG; *n* = 11), NTG (*n* = 6), pigment glaucoma (PG; *n* = 2), angle-closure glaucoma (ACG; *n* = 3), and unclassified glaucoma (*n* = 2). The remaining 30 eyes were non-glaucomatous controls. The main patient characteristics and OCT/OCT-A measurements (Table 1; Supplementary Tables S1 and S2) were consistent with our previous study [13]. We found no significant differences between the control and glaucoma groups in terms of age, sex, pseudophakia, or MAP. However, the glaucoma group showed elevated IOP, better visual acuity, increased vertical CDR and cup volume, and reduced GCC thickness, RNFL thickness, rim area, and ONH VD, as well as reduced VD in the macular area and the perifoveal superficial vascular area.

### 3.2. Peripheral and Intraocular Cytokine Profile

The plasma levels of BMP-9 and the AqH levels of CNTF, angiopoetin-1, arginase-1, and BMP-9 were rarely detected and were thus excluded from further analysis. The plasma levels of endostatin were above the upper limit of quantification and were therefore also excluded. For the plasma levels of IL-6, IL-8, sLOX-1, NTproBNP, PDGF-BB, and survivin, and for the AqH levels of BigET-1, IL-6, NTproBNP, BMP-2, and sE-selectin, only Fisher’s exact test or the chi-square test was applied, as only outliers were present (see Section 2).

**Figure 1:**
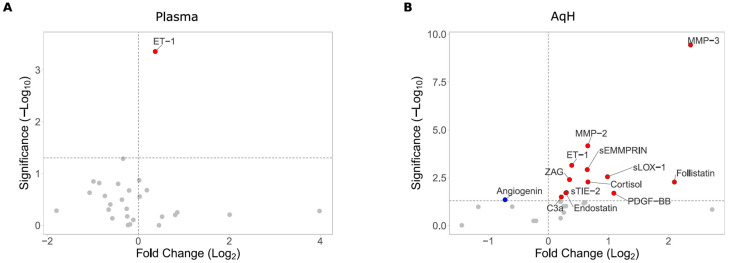
Cytokine levels in (**A**) plasma and (**B**) AqH of glaucoma versus participants with cataract (Supplementary Table S3) are visualized in volcano plots. The volcano plots showing the Log2-Fold change (FC) value (x-axis; factor of the mean protein pg/mL in glaucoma divided by the mean pg/mL in cataract) and −Log10 *p*-value (y-axis; negative Log10 calculated from the unadjusted *p*-values). In each graph, every point represents an individual protein. The vertical lines represent Log2-FC ≥ 2, both upregulated (right side) and downregulated (left side), and the horizontal lines represent a −Log10 *p*-value ≥ 1.3 (equal to *p*-value *p* < 0.05) as the threshold cutoff. The significantly altered proteins are labeled: red circle indicates significantly elevated protein level in glaucoma (−Log10 <1.3; *p* < 0.05); blue circle indicates significantly reduced protein level in glaucoma (−Log10 < 1.3; *p* < 0.05); gray circle indicates cytokines showing no significant difference between the two groups.

As previously demonstrated [13], plasma (adjusted *p* = 0.0016) and AqH (adjusted *p* = 0.0022) samples showed significantly elevated ET-1 levels in glaucoma participants compared with controls (*p* < 0.01) (Figure 1, Supplementary Figure S1, Supplementary Table S3). Apart from the elevated plasma ET-1 levels, no significant differences in cytokine levels were observed between glaucoma and control participants (Figure 1A; Supplementary Table S3).

Luminex analysis of AqH samples revealed significantly elevated levels of MMP-2, MMP-3, ET-1, sEMMPRIN, ZAG, sLOX-1, follistatin, cortisol, endostatin, sTIE-2, and PDGF-BB in glaucoma participants (adjusted *p* < 0.05) (Figure 1B; Supplementary Figure S1; Supplementary Table S3). Elevated C3a and lower angiogenin levels (unadjusted *p* < 0.05) did not reach statistical significance after Benjamini–Hochberg adjustment (Supplementary Table S3).

Within the glaucoma group, no correlation was observed between the differentially expressed AqH cytokines and their peripheral plasma levels (Supplementary Table S6).

### 3.3. Correlation of Peripheral and Intraocular Cytokines with Clinical Parameters

Consistent with our previous study [13], in the total cohort (glaucoma and control), the ET-1 plasma levels exhibited a moderate positive correlation with GLV (r = 0.321) and cup volume (r = 0.313), and a negative correlation with GCC thickness (r = −0.333) and with the VD of PeriONH TempSup (r = −0.382) and PeriONH TempInf (r = −0.359) (Supplementary Table S4). In the total cohort, none of the measured plasma cytokines showed a correlation with age, IOP, or GSS (Supplementary Table S4). An elevated ET-1 level in AqH showed a moderate positive correlation with cup volume (r = 0.313) and a negative correlation with the RNFL superior (r = −0.359) and with the VD of PeriONH NasSup (r = −0.338) (Supplementary Table S4). AqH PlGF-1 level correlated moderately with IOP (r = 0.315), AqH sLOX-1 level correlated moderately with age (r = 0.325), AqH MMP-2 level correlated moderately with the first diagnosis of glaucoma (r = 0.314), and AqH MMP-3 level correlated moderately with visual acuity (r = −0.333) in the total cohort (Supplementary Table S4). Furthermore, moderate negative correlations of AqH, APN, C3a, MMP-2, MMP-3, and ZAG levels with the reduced VD could be observed in several areas of the ONH region (rho < −0.3). The most negative correlation was found between AqH MMP-3 levels and VD of several PeriONH sectors, and additionally with the VD of several PeriFovea SVP sectors (r < −0.3). Furthermore, MMP-2 and MMP-3 correlated moderately with the CDR (rho > 0.3) and rim area (rho < −0.3) (Supplementary Table S4).

In the glaucoma cohort, plasma ET-1 levels showed a negative correlation with the VD of the PeriONH TempSup area (r = −0.341) and with the fovea of the SVP/DVP area (r = −0.339/r = −0.357). Plasma Big-ET-1 level correlated moderately with the IOP (r = 0.312). Further, levels of APN, sLOX-1, and resistin in AqH correlated moderately with age (rho > 0.3; Supplementary Table S5).

Within the glaucoma group, APN, C3a, and ZAG showed the most correlations with reduced VD in the PeriONH area (rho > 0.3). MMP-3, resistin, and sTIE-2 AqH levels correlated with reduced VD in a few PeriONH sectors (rho < −0.3; Supplementary Table S5), while the PDGF-BB levels in AqH showed a positive correlation with the VD in the Fovea SVP area (r = 0.329; Supplementary Table S5).

### 3.4. Multivariable Analysis (MVA)

To identify potential glaucoma-associated factors, all clinical OCT/OCT-A parameters, as well as ET-1 and cytokine levels in AqH/plasma samples, were utilized in a multivariable setting (Supplementary Table S7). GCC, RNFL, score tool, OCT VD PeriONH Average, and VD Macula SVP Whole were defined as dependent variables. All other clinical and soluble parameters were included as independent variables, and their possible correlation with the defined dependent variables were analyzed in MVA. The statistical correlation of variables (independent) to a target variable (dependent) is necessary but not sufficient to identify risk factors; thus, significant correlating independents are potential clinical risk factors or biomarkers for the targets.

We applied the MVA to the complete data set and to the glaucoma cases (Table 2; Supplementary Table S8). Because the glaucoma group is smaller than the complete set, the model size and the achieved pseudo-R^2^ are lower for the glaucoma group.

For small pseudo-R^2^ (<0.5), the multivariable model is less reliable, and the results had to be validated using univariable analysis. The morphological biomarkers specific for glaucomatous changes in RNFL, GCC, GLV, and CDR are the most dominant factors of each MVA. The further specific factors that occurred at least two times are: topical beta-blockers (significant influence on VD PeriONH and GSS Score), APN AqH (significant influence on VD PeriONH), MMP-9 plasma (significant influence on GCC and GSS Score), and sE-Selectin plasma (significant influence on RNFL thickness). The specific factors in the AqH that occurred once are MMP-2, sTIE-2, endoglin, ZAG, and angiostatin.

## 4. Discussion

Emerging evidence suggests that distinct intraocular protein patterns, including the vasoconstrictor ET-1, contribute to glaucoma development and progression through regulation of AqH outflow, modulation of IOP, and influence on OP. These altered protein profiles can lead to ischemia, oxidative stress, and neuroinflammation, ultimately resulting in RGC death and optic nerve damage [13,39,40,41,42].

This study comprehensively analyzed levels of various cytokines and ET-1 in the AqH and blood plasma samples of participants with OAG, examining their correlations with clinical parameters. In the following discussion, we focus on statistically significant results or important factors that emerged in the multivariable analysis.

### 4.1. ET-1

ET-1 acts as a counterplayer to nitric oxide (NO) in the regulation of vascular tone and AqH dynamics [43]. While NO lowers IOP by relaxing trabecular meshwork cells [44], ET-1 causes vasoconstriction and impairs aqueous outflow [43]. Elevated ET-1 levels in glaucoma AqH suggest an imbalance favoring vasoconstriction, contributing to increased outflow resistance, elevated IOP, and compromised ONH perfusion [43,45]. Our previous study [13] identified elevated plasma ET-1 levels as a biomarker for impaired OP. The current findings extend this by demonstrating correlations between ET-1 and both vascular and structural parameters. Animal models have shown that inhibiting ET-1 activity can lower IOP and protect RGCs [46,47,48], supporting the therapeutic potential of targeting the NO-ET-1 axis. Due to the limited volume of AqH samples, we could not directly measure NO levels, which would have provided valuable insights into the NO–ET-1 balance and remains an important area for future investigation.

### 4.2. Matrix Metalloproteinases

MMPs and their regulators are widely expressed in various ocular tissues including the trabecular meshwork [49,50].

As proteolytic enzymes, MMPs modulate the ECM of the trabecular meshwork, thereby influencing ocular outflow resistance and IOP [50]. Beyond their role in maintaining the classical outflow pathway, MMPs are expressed in various neurons and glial cells of the retina and optic nerve. Increased MMP activity may contribute to RGC degeneration by degrading structural components of the ECM in retinal tissue, leading to increased permeability of the blood-retina barrier and enhanced neuroinflammation [51]. Several studies have demonstrated elevated MMP-2 and MMP-3 levels in the AqH of patients with POAG [23,24,52,53]. Our results confirm elevated MMP-2 and MMP-3 levels in glaucoma AqH. The role of MMPs in glaucoma is further strengthened by the elevated intraocular sEMMPRIN levels observed in the current study, an inducer of MMPs [49].

MMP-3 also plays an important role in free fatty acid-induced insulin resistance and angiogenesis by regulating the secretion of TNFα and VEGF. Its contribution to the development of insulin resistance and type 2 diabetes has been discussed [54]. Our results suggest that systemic MMP-9 may still play a role in glaucoma pathogenesis despite normal AqH levels.

### 4.3. Additional Significant Biomarkers

The elevated **follistatin** levels observed in our study may represent a compensatory response to counteract TGF-β2 activity in glaucomatous eyes [55], suggesting active regulation of fibrotic pathways in the trabecular meshwork and retina.

Elevated levels of the angiogenesis inhibitors **endostatin** might point to an impairment of vascular supply and nutrient delivery to the ONH, as previously shown for endostatin in POAG [56].

**PDGF-BB,** a growth factor expressed by RGC, has been shown to exert autologous neuroprotective effects [57]. The present study showed positive correlations of PDGF-BB with OP parameters, pointing to a potential neuroprotective role in the pathogenesis of glaucoma.

**sTIE-2,** which correlated with impaired OP, appeared as a negative biomarker for RNFL thickness. As a soluble form of the angiopoietin receptor Tie-2, elevated sTIE-2 may interfere with normal angiopoietin signaling and vascular stability [58], though its specific role in glaucoma-related vascular dysfunction requires further investigation.

**LOX-1** is a receptor for oxidized LDL involved in endothelial dysfunction and atherosclerosis. Elevated serum sLOX-1 levels serve as a biomarker for cardiovascular diseases [59,60]. Though the elevated LOX-1 AqH levels in our study did not correlate with clinical glaucomatous parameters, it points to a systemic vascular involvement in glaucoma pathogenesis.

The altered AqH levels of **C3a**, **cortisol**, and **angiogenin** observed in our glaucoma cohort are consistent with the findings of previous studies [61,62,63].

However, these alterations did not correlate with any of the clinical parameters measured in our study and might suggest that their role in glaucoma pathogenesis may be more complex than direct associations with structural or functional damage.

### 4.4. Metabolic Regulators: APN, ZAG, and Resistin

A central finding of our study is the identification of metabolic biomarkers (APN, ZAG, and resistin) associated with impaired OP. A possible link between glaucoma and diabetes/insulin resistance, which is currently a topic of frequent discussion in the literature [64]. Notably, these findings emerged unexpectedly from our non-diabetic cohort, as diabetes mellitus was an exclusion criterion in our study design to eliminate potential confounders affecting OP measurements.

**APN** emerged as potential biomarker of reduced OP. APN has anti-inflammatory, anti-oxidative, insulin-sensitizing, angiogenic, and vasodilatory properties, promoting glucose uptake, fatty acid oxidation, and insulin sensitivity. Low APN levels are typical for insulin resistance and type 2 diabetes [65]. The correlation between APN and impaired OP suggests that APN may play a role in the regulation of OP via its vasodilatory and angiogenic properties, influencing the microcirculation around the ONH. Interestingly, a positive effect of ET-1 on APN release has been demonstrated [66], suggesting a complex feedback mechanism.

**ZAG** is involved in lipid degradation, fatty acid mobilization, and immune responses [67]. It enhances insulin sensitivity by facilitating glucose uptake through the induction of insulin receptor 1 and APN [68]. Elevated ZAG and reduced insulin degrading enzyme levels have been detected in the tear fluid of patients with glaucoma [69]. The increased ZAG levels in our study may indicate modulation of insulin sensitivity in the ocular environment.

**Resistin** is also discussed in the context of IR [70], where AqH levels are correlated with the impaired OP in glaucomatous eyes in the current study. Insulin resistance could lead to the impairment of energy metabolism and function of RGC, contributing to glaucoma development [28]. The emergence of these metabolic markers in our non-diabetic participants is particularly intriguing, suggesting that subclinical metabolic dysfunction may contribute to glaucoma pathogenesis even in the absence of overt diabetes. While we acknowledge the limitation of not measuring systemic metabolic parameters (glucose, insulin, HOMA-IR) in our non-diabetic cohort, our findings align with the hypothesis of metabolic dysfunction in glaucoma proposed by Faiq et al. [27] and highlight the potential of these metabolic markers as biomarkers for metabolic stress in glaucomatous eyes. Future studies should include comprehensive metabolic profiling even in non-diabetic participants to better understand these relationships.

### 4.5. Plasma Biomarkers and Systemic Involvement

As previously shown, elevated peripheral plasma **ET-1** level [13], correlated most often with glaucomatous clinical parameters. Besides ET-1, other peripheral cytokine levels showed no significant differences. However, a correlation of peripheral **sE-Selectin plasma** and **MMP-9** with glaucomatous changes are shown in the MVA. These findings suggest that certain plasma markers may reflect systemic processes contributing to glaucoma, even when AqH levels are not elevated.

### 4.6. Integration of Biomarkers into Clinical Practice

A practical challenge is that many significant biomarker alterations were detected in AqH, requiring anterior chamber paracentesis for sample collection. While this procedure carries minimal risk when performed by experienced ophthalmologists, it remains invasive. For routine applications, sampling could be performed during scheduled surgical procedures such as cataract surgery or glaucoma interventions. For risk stratification in selected high-risk patients or those with unexplained progression despite IOP control, a dedicated anterior chamber tap may be justifiable given the potential benefits of targeted therapy.

Despite this limitation, the integration of these biomarkers into clinical practice could address several current challenges in glaucoma management. Currently, treatment decisions rely primarily on IOP measurements and structural changes, which often indicate that irreversible damage has already occurred. The identified biomarkers could enable earlier detection, as metabolic and vascular alterations reflected by ET-1, APN, and ZAG may precede detectable structural damage, allowing intervention before significant RGC loss occurs.

Understanding individual pathophysiological profiles through biomarker analysis could guide personalized treatment approaches, rather than the current “one-size-fits-all” IOP-lowering strategy. This would allow clinicians to distinguish between patients with primarily vascular dysfunction, metabolic stress, or inflammatory processes and tailor therapy accordingly. These biomarkers could also improve risk stratification by identifying patients with elevated biomarker levels despite normal IOP as high-risk individuals, warranting closer monitoring and potentially earlier treatment initiation.

Most importantly, patients who continue to progress despite IOP control could be identified through persistent biomarker elevation, prompting alternative therapeutic strategies before further irreversible damage occurs. For such high-risk patients or those with unexplained progression, targeted biomarker analysis obtained through dedicated anterior chamber sampling could guide personalized therapeutic strategies beyond conventional IOP reduction, with the procedure being justifiable given the potential benefits of targeted therapy.

Translation into clinical practice would require establishing reference ranges in larger populations, developing standardized assay protocols, conducting prospective validation studies, and performing cost-effectiveness analyses. Future research should explore correlations between AqH and more accessible body fluids to develop less invasive screening methods. Given glaucoma’s heterogeneous nature, a panel approach combining multiple biomarkers with imaging parameters will likely prove more valuable than single markers alone.

## 5. Limitations

Due to the single-center study design and the relatively small sample size in the subgroups of different types of glaucoma, the generalizability of the results is restricted. The study included NTG participants in the glaucoma group, while the others had high intraocular pressure. Additionally, our cohort included patients with XFG, both of which have been associated with prominent vascular dysfunction as part of their pathogenic mechanisms. While recent OCT-A studies demonstrated similar reductions in optic disc perfusion between NTG and POAG patients [71], suggesting shared vascular alterations across the glaucoma spectrum, the heterogeneity of glaucoma subtypes with varying degrees of vascular involvement may have influenced our biomarker findings. We deliberately retained NTG and XFG participants in our analysis, as these represent clinically important glaucoma subtypes. Their exclusion based solely on vascular pathogenic components would have artificially narrowed our study scope, particularly given that vascular dysfunction is increasingly recognized across all glaucoma subtypes.

A correlation between topical beta-blocker uses and glaucomatous changes suggests that topical glaucoma medications may influence OP. Future studies should address this question to improve our understanding of the potential impact of glaucoma treatment on the pathophysiology of the disease.

The study has an exploratory character, as a multitude of cytokines and clinical parameters were analyzed. Due to data size limitation, not all independent variables can be analyzed simultaneously in one multivariable model. Thus, the model size is restricted by using Lasso as a variable selection method, thereby important variables may have been excluded.

Although significant correlations were found between various cytokines and clinical as well as OCT/OCT-A parameters, the cross-sectional analysis does not permit conclusions about causality. Therefore, the results should be considered as hypothesis-generating and should be verified in further confirmatory studies. Longitudinal studies are necessary, with repeated measurements, to investigate the temporal dynamics and possible causal relationships.

Some of the results found in this study suggest a possible link between glaucoma and diabetes/insulin resistance The study did not directly measure metabolic factors such as blood glucose and insulin levels in our non-diabetic cohort, limiting our ability to fully explore the metabolic dysfunction hypothesis that emerged from our findings. Future studies should include comprehensive metabolic profiling even in non-diabetic participants to better understand the role of subclinical metabolic dysfunction in glaucoma pathogenesis.

## 6. Conclusions

This study demonstrates a strong association between impaired OP and specific biomarker patterns in glaucoma patients, pointing to three key pathophysiological axes that may contribute to glaucoma progression:

First, the **vascular axis** confirms ET-1’s dual role in vascular dysfunction and neurodegeneration in glaucoma.

Second, the **extracellular matrix remodeling axis** showing the most extensive correlations with clinical parameters, impaired OP, and structural changes in glaucoma.

Third, and most unexpectedly, a **metabolic axis** emerging from our non-diabetic cohort may contribute to glaucoma pathogenesis even in the absence of overt diabetes.

## Figures and Tables

**Table 1:** Clinical data and OCT/OCT-A parameters.

	Control	Glaucoma	Adj. *p*-Value	
Clinical Data				
Glaucoma Entity (*n*)	POAG:0| XFG:0| NTG:0| PG:0| ACG:0	POAG:63| XFG:11| NTG:6| PG:2| ACG:3| NA:2	N.A.	
1st Diagnosis of Glaucoma (Mo)	N.A.	62.00 [27.50–120.00]	N.A.	
Age at Operation	66.77 ± 9.01	67.00 [57.00–73.00]	0.8395	^2^
Sex (*n*)	Female:20| Male:10	Female:48| Male:39	0.4622	^1^
IOP (mmHg)	14.00 [12.00–14.00]	18.00 [15.00–23.50]	<0.00001 *	^3^
Refractive Error (D)	0.07 ± 2.88	0.00 [−1.50–0.75]	0.3477	^3^
Visual Acuity (LogMAR)	0.40 [0.30–0.60]	0.20 [0.02–0.30]	<0.00001 *	^3^
MAP (mmHg)	95.00 [92.50–99.50]	95.00 [91.67–101.17]	0.88884	^3^
Topical Antiglaucoma	0 [0–0]	3.00 [1.00–3.50]	N.A.	
Beta-blocker, *n* (%)	(0%)	53 (60.9%)	N.A.	
Carbonic Anhydrase Inhibitor, *n* (%)	(0%)	58 (66.7%)	N.A.	
Prostaglandin Analog. *n* (%)	(0%)	64 (73.6%)	N.A.	
Alpha-adrenergic Agonist, *n* (%)	(0%)	28 (32.2%)	N.A.	
Pilocarpine, *n* (%)	(0%)	2 (2.3%)	N.A.	
Systemic Carbonic Anhydrase Inhibitor, *n* (%)	(0%)	6 (8.6%)	N.A.	
OCT and OCT-A Parameters				
Ganglion Cell Complex (µm)	96.55 ± 8.41	77.00 [69.25–86.00]	<0.00001 *	^3^
Focal Loss Volume (%)	0.41 [0.21–0.80]	5.48 [2.17–9.12]	<0.00001 *	^3^
Global Loss Volume (%)	2.10 [0.50–4.25]	17.97 ± 10.73	<0.00001 *	^3^
RNFL Thickness (µm)	96.79 ± 8.72	74.32 ± 13.87	<0.00001 *	^2^
Cup/Disc Ratio Total	0.31 ± 0.15	0.65 [0.55–0.76]	<0.00001 *	^3^
Rim Area (mm^2^)	1.37 ± 0.35	0.73 [0.52–0.96]	<0.00001 *	^3^
Disc Area (mm^2^)	2.03 ± 0.32	2.08 ± 0.36	0.562	^2^
VD ONH Whole (%)	47.00 ± 2.52	35.97 ± 6.86	<0.00001 *	^3^
VD Macula SVP Whole (%)	41.90 ± 4.15	37.39 ± 4.40	<0.00001 *	^2^
VD Fovea SVP (%)	19.99 ± 6.69	17.85 [11.22–21.95]	0.128	^3^
VD Macula DVP Whole (%)	39.96 ± 4.37	41.79 ± 4.75	0.124	^2^
VD Fovea DVP (%)	34.28 ± 8.49	32.39 ± 8.08	0.380	^2^
FAZ (mm^2^)	0.26 ± 0.10	0.25 [0.19–0.35]	0.549	^3^

N.A. not applicable; POAG, primary open-angle glaucoma; XFG, exfoliative glaucoma; NTG, normal-tension glaucoma; PG, pigmentary glaucoma; ACG, angle-closure glaucoma; MAP, mean arterial pressure; RNFL, retinal nerve fiber layer; ONH, optic nerve head; VD, vessel density; FAZ, foveal avascular zone; SVP, superficial vascular plexus; DVP, deep vascular plexus. ^1^ indicates Chi-square-test; ^2^ indicates *t*-test; ^3^ indicates Wilcoxon rank sum test; * indicates *p* < 0.05.

**Table 2:** Multivariable Analysis.

Dependent Variables	Group	*n*	Residual Deviance	Null Deviance	Pseudo R^2^	Independent Variables
**VD PeriONH Average**	**Glaucoma**	63	1590.6	4603.1	0.65	RNFL (+) APN AqH (−) VCDR (−)
	**All**	86	1593.8	7799.1	0.80	RNFL (+) APN AqH (−) ZAG Plasma (+) VCDR (−) MMP-2 AqH (−) Topical Beta-Blocker (−)
**OCT GSS Score**	**Glaucoma**	68	136.88	205.78	0.33	Ganglion Cell Complex (−) Topical Beta-Blocker (+)
	**All**	94	79.707	319.29	0.75	Global Loss Volume (+) Age at Operation (−) MICB plasma (−) Topical Beta-Blocker (+) Pseudophakia (+) MMP-9 Plasma (+)
**RNFL Thickness**	**Glaucoma**	69	3053.2	13,412.6	0.77	Ganglion Cell Complex (+) sE-Selectin Plasma (+) sTIE-2 AqH (−)
	**All**	93	4524.6	23,651.2	0.81	Global Loss Volume (−) Ganglion Cell Complex (+) sE-Selectin Plasma (+) Group MMP-3 AqH
**Ganglion Cell Complex**	**Glaucoma**	69	3496.5	13,212.6	0.74	RNFL (+) Endoglin AqH (+) Follistatin Plasma Total Cup/Disc Ratio
	**All**	86	3296.4	17,593.6	0.81	RNFL (+) VCDR (−) Angiostatin AqH (−) ZAG AqH (+) MMP-9 Plasma (+) FAZ (−) PIGF-1 Plasma
**VD Macula SVP Whole**	**Glaucoma**	65	784.54	1179.39	0.33	RNFL (+) VEGF-A Plasma (+) Rim Area
	**All**	86	742.19	1926.89	0.61	Global Loss Volume (−) Rim Area (+) Sex = Male (−) FAZ-AI (−) VEGF-A Plasma APN AqH MMP-9 Plasma

Pseudo-R^2^ computed by McFadden’s method; (+) positive or (−) negative significant correlation between a certain independent variable and a dependent variable. Complete results are summarized in Supplementary Table S8.

## Data Availability

The raw data supporting the conclusions of this article will be made available by the authors on request.

## Supplementary Materials

The following supporting information can be downloaded at: https://www.mdpi.com/article/10.3390/biom15101364/s1, Figure S1: ET-1 level [pg/mL] in plasma and AqH samples from controls and patients participants with glaucoma; Figure S2: AqH levels of MMP-2, MMP-3, LOX-1, TIE-2, follistatin, sEMMPRIN, ZAG, C3a, cortisol, angiogenin, and APN [pg/mL] from controls and participants with glaucoma; Table S1: Clinical data, OCT-A parameters, and ET-1 level; Table S2: OCT and OCT-A parameters; Table S3: Plasma and AqH ET-1 and cytokine levels; Table S4: Correlation of plasma and AqH ET-1 and cytokine level with clinical data and OCT/OCT-A parameter (all); Table S5: Correlation of plasma and AqH ET-1 and cytokine level with clinical data and OCT/OCT-A parameter (glaucoma); Table S6: Correlation of plasma and corresponding AqH cytokine level (glaucoma) Table S7: Benjamini–Hochberg on Glaucoma vs. Control; Table S8: Multivariable analysis and p-adjustment.

## Abbreviations

ACG	angle-closure glaucoma
AI	acircularity index
APN	adiponectin
AqH	aqueous humor
DVP	deep vascular plexus
ET-1	endothelin-1
FAZ	foveal avascular zone
GCC	ganglion cell complex
GLV	global loss volume
ILM	internal limiting membrane
IOP	intraocular pressure
IPL	inner plexiform layer
IR	insulin resistance
MAP	mean arterial pressure
MVA	multivariable analysis
NO	nitric oxide
NTG	normal-tension glaucoma
OCT	optical coherence tomography
OCT-A	optical coherence tomography angiography
ONH	optic nerve head
OP	ocular perfusion
OPL	outer plexiform layer
PG	pigmentary glaucoma
PGA	prostaglandin analogous
POAG	primary open-angle glaucoma
RGC	retinal ganglion cell
RNFL	retinal nerve fiber layer thickness
SVP	superficial vascular plexus
VCDR	vertical cup-to-disc ratio
VD	vessel density
XFG	pseudoexfoliation glaucoma
