# Supplementary material for: ET-1, MMPs, ZAG, and APN Link Reduced Ocular Perfusion to Glaucoma
Source: Biomolecules. 2025 Sep 25;15(10):1364. doi: 10.3390/biom15101364 (PMC12562824; doi:10.3390/biom15101364)
Supplement: Supplementary file 1 [file biomolecules-15-01364-s001.zip › biomolecules-3766285-Supplementary Figures.pdf]

Supplementary Figures

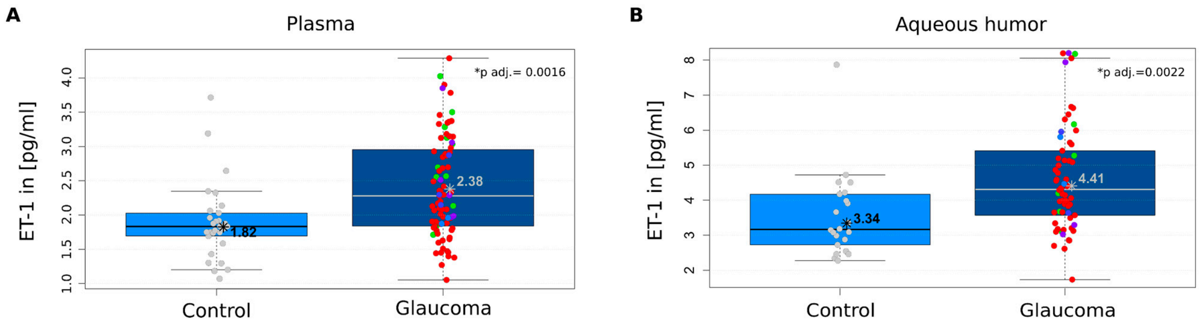

Figure S1. ET-1 level [pg/mL] in plasma and AqH samples from controls and patients participants with glaucoma.

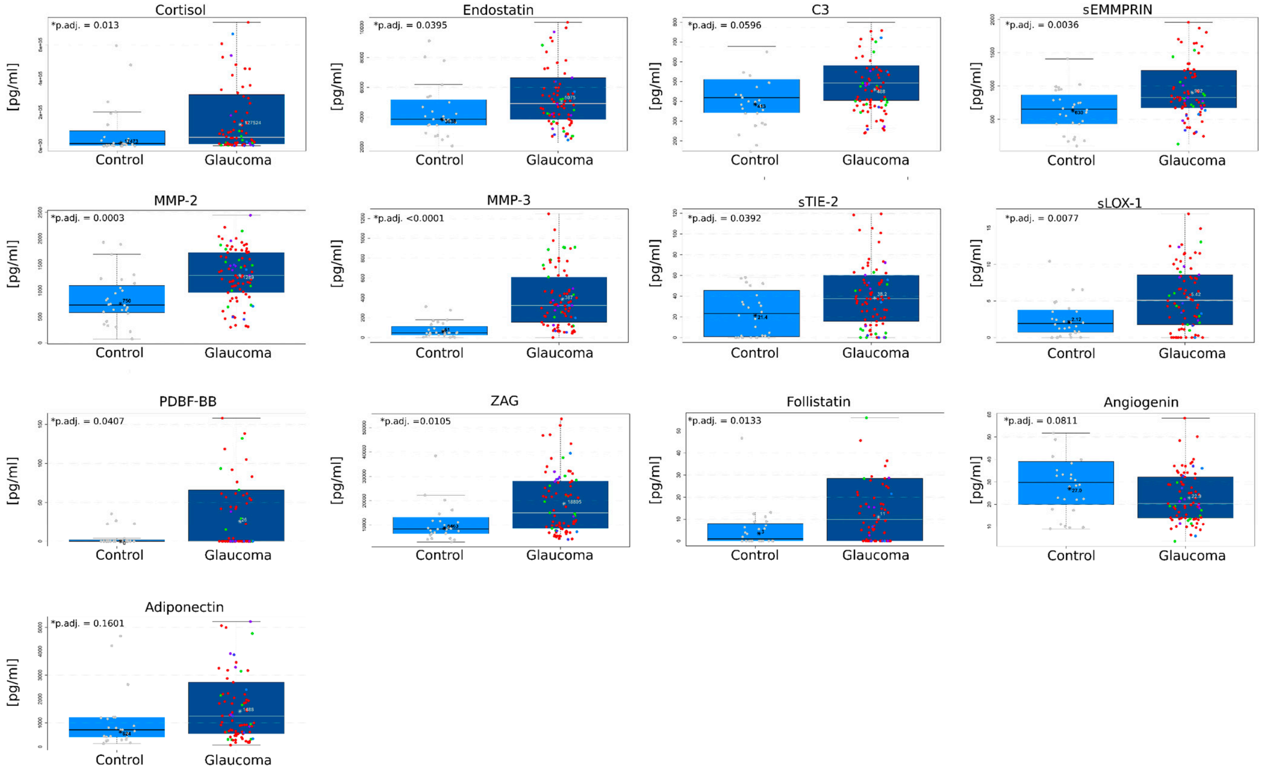

Figure S2. AqH levels of MMP-2, MMP-3, LOX-1, TIE-2, follistatin, sEMMPRIN, ZAG, C3a, cortisol, angiogenin, and APN [pg/mL] from controls and participants with glaucoma.
